# Supplementary material for: Immunosuppressive regimens based on Cyclophospamide or Calcineurin inhibitors: Comparison of their effect in the long term outcome of Primary Membranous Nephropathy
Source: PLoS One. 2019 Aug 12;14(8):e0217116. doi: 10.1371/journal.pone.0217116 (PMC6690570; doi:10.1371/journal.pone.0217116)
Supplement: S1 File — (DOCX) [file pone.0217116.s001.docx]

**Supplementary Information**

Participating Hospitals/Institutions

^1^Department of Nephrology, Hippokration General Hospital, Aristotle University, Thessaloniki, Greece,

^2^Department of Nephrology, Laiko General Hospital, National and Kapodistrian University, Athens, Greece,

^3^Department of Nephrology, University Hospital of Patras, Patras, Greece, ^4^Department of Nephrology, General Hospital of Nikaia, Piraeus, Greece,

^5^Department of Nephrology, University Hospital of Ioannina, Ioannina, Greece,

^6^Department of Nephrology, Hatzikosta General Hospital of Ioannina, Ioannina, Greece,

^7^Department of Nephrology, Papageorgiou General Hospital of Thessaloniki, Thessaloniki, Greece,

^8^Department of Nephrology, Evangelismos General Hospital, Athens, Greece,

^9^Department of Nephrology, Gennimatas General Hospital of Athens, Athens, Greece,

^10^Department of Nephrology, University Hospital of Heraklion, Heraklion Crete, Greece,

^11^Department of Nephrology, University Hospital of Larissa, Larissa, Greece,

^12^Department of Nephrology, University Hospital of Alexandroupolis, Alexandroupoli, Greece

^13^ Department of Nephrology, Tzaneion General Hospital of Piraeus, Athens, Greece

^14^Department of Nephrology, Venizelio General Hospital of Heraklion, Heraklion Crete, Greece,

^15^Department of Nephrology, General Hospital of Chania, Chania Crete, Greece,

^16^ Section of Nephrology, 1st Department of Medicine, AHEPA University General Hospital, Thessaloniki, Greece

^17^Department of Nephrology, Attikon University Hospital, National and Kapodistrian University, Athens, Greece

^18^Department of Nephrology, Aretaieio Hospital, National and Kapodistrian University of Athens, Athens, Greece

^19^Department of Nephrology, General Hospital of Thessaloniki "Agios Pavlos", Thessaloniki, Greece

^20^Department of Nephrology, Hellenic Red Cross Hospital Korgialeneio-Benakeio, Athens, Greece

^21^Department of Nephrology, General Hospital of Rhodes, Rhodes, Greece

^22^ Renal Unit, General Hospital of Drama, Drama, Greece

^23^Department of Nephrology, General Hospital of Kavala, Kavala, Greece
